# LP-UV-Nano MgO_2_ Pretreated Catalysis Followed by Small Bioreactor Platform Capsules Treatment for Superior Kinetic Degradation Performance of 17α-Ethynylestradiol

**DOI:** 10.3390/ma13010083

**Published:** 2019-12-23

**Authors:** Lakshmi Prasanna Vaddadi, Dror Avisar, Vinod Kumar Vadivel, Ofir Menashe, Eyal Kurzbaum, Vered Cohen-Yaniv, Hadas Mamane

**Affiliations:** 1 The Water Research Center, Hydrochemistry Laboratory, Porter School for Environment and Earth Sciences, Raymond and Beverly Sackler Faculty of Exact Sciences, Tel Aviv University, Tel Aviv 69978, Israel; lakshmip@mail.tau.ac.il; 2 The Water Research Center, Environmental Engineering Program, School of Mechanical Engineering, Faculty of Engineering, Tel Aviv University, Tel Aviv 69978, Israel; vinodkumarv@mail.tau.ac.il (V.K.V.); verver812@gmail.com (V.C.-Y.); hadasmg@tauex.tau.ac.il (H.M.); 3 Water Industry Engineering Department, Achi Racov Engineering School, Kinneret College on the Sea of Galilee, M.P. Emek Ha’Yarden 15132, Israel; 4 Shamir Research Institute, University of Haifa, P.O. Box 97, Qatzrin 12900, Israel; ekurzbaum@univ.haifa.ac.il; 5 Department of Geography and Environmental Studies, University of Haifa, Mount Carmel, Haifa 3498838, Israel

**Keywords:** 17α-ethynylestradiol (EE2), nano MgO_2_, LP-UV photocatalysis, biodegradation, small bioreactor platform (SBP)

## Abstract

A successful attempt to degrade synthetic estrogen 17α-ethynylestradiol (EE2) is demonstrated via combining photocatalysis employing magnesium peroxide (MgO_2_)/low-pressure ultraviolet (LP-UV) treatment followed by biological treatment using small bioreactor platform (SBP) capsules. Reusable MgO_2_ was synthesized through wet chemical synthesis and extensively characterized by X-ray diffraction (XRD) for phase confirmation, X-ray photoelectron spectroscopy (XPS) for elemental composition, Brunauer-Emmett-Teller (BET) to explain a specific surface area, scanning electron microscopy (SEM) imaging surface morphology, and UV-visible (Vis) spectrophotometry. The degradation mechanism of EE2 by MgO_2_/LP-UV consisted of LP-UV photolysis of H_2_O_2_ in situ (produced by the catalyst under ambient conditions) to generate hydroxyl radicals, and the degradation extent depended on both MgO_2_ and UV dose. Moreover, the catalyst was successfully reusable for the removal of EE2. Photocatalytic treatment by MgO_2_ alone required 60 min (~1700 mJ/cm^2^) to remove 99% of the EE2, whereas biodegradation by SBP capsules alone required 24 h to remove 86% of the EE2, and complete removal was not reached. The sequential treatment of photocatalysis and SBP biodegradation to achieve complete removal required only 25 min of UV (~700 mJ/cm^2^) and 4 h of biodegradation (instead of >24 h). The combination of UV photocatalysis and biodegradation produced a greater level of EE2 degradation at a lower LP-UV dose and at less biodegradation time than either treatment used separately, proving that synergetic photocatalysis and biodegradation are effective treatments for degrading EE2.

## 1. Introduction

Endocrine-disrupting chemicals (EDCs) are emerging contaminants that pose a serious threat to the environment, humans, and livestock. It has been estimated that an average of 30,000 kg of natural estrogens, 700 kg of synthetic hormones from humans, and 83,000 kg of estrogens from livestock are released annually into the environment [1]. The presence of estrogens in wastewater-treatment plants (WWTPs) has been reported in many countries, such as Brazil, Canada, and Italy, implying that the methods employed in WWTPs do not degrade estrogens. Hence, new methods should be adapted to degrade micropollutants such as EDCs [2]. To the best of our knowledge, there is no cost-effective treatment barrier to prevent chronic exposure to EDCs. The synthetic EDC 17α-ethynylestradiol (EE2), widely used in oral contraceptives and hormone-replacement therapy, has the potential to elicit negative effects in the endocrine systems of humans and wildlife. Advanced oxidation processes (AOPs) are used to oxidize/mineralize organic pollutants in water through the accelerated production of hydroxyl free radical (·OH). AOPs such as ozonation [3], ultraviolet (UV)/hydrogen peroxide (H_2_O_2_) treatment [4], and Fenton and photo-Fenton degradation [5,6] have been adopted to remove EE2 from water. UV/H_2_O_2_ is one of the most investigated AOP techniques for the removal of organic contaminants from wastewater [7]. However, H_2_O_2_ slowly decomposes to oxygen and water, and therefore needs to be continuously added to the water stream. Consequently, a catalyst that can produce H_2_O_2_ in situ would be beneficial for real-time applications.

Solid metal peroxides, such as ZnO_2_, MgO_2_, and CaO_2_, can produce H_2_O_2_ when suspended in water, and conditions such as pH and temperature affect H_2_O_2_ production [8]. The Fenton-like degradation of organic contaminants has been investigated using metal peroxides as the H_2_O_2_ source. Even though Fenton-like degradation is efficient, it is effective only at acidic pH, and the formation of excess iron sludge during the process requires further treatment to remove the sludge, resulting in additional operational costs.

In bioaugmentation, selected microbial flora is added to the system to accelerate the removal of organics, heavy metals, nutrients, and others. Even though the technique is efficient in the degradation of organic and inorganic contaminants, the maintenance of a sufficient amount of biomass culture in the water body, especially in a continuous-treatment model where the entire water body is exchanged every few hours resulting in culture dilution, remains a big challenge. Menashe and Kurzbaum [9] used small bioreactor platform (SBP) technology to encapsulate bacteria inside 3D capsules composed of cellulose-acetate membranes. Capsules added to a domestic mixed liquor protected the biomass from negative interactions with the natural biota and prevented culture dilution in the host bioreactor. The encapsulated bacteria were able to improve bioreactor treatment yield for domestic wastewater and protect the treatment process from stress episodes.

Azaizeh et al. [10] demonstrated the efficiency of an autochthonous bacterial consortium from olive mill wastewater in phenol biodegradation, while Kurzbaum et al. [11] demonstrated phenol biodegradation using SBP-encapsulated *Pseudomonas putida* F1. The phenol-biodegradation rate of the suspended form and the SBP-encapsulated form were similar, demonstrating the potential of the SBP technology. Oz et al. [12] also demonstrated the efficiency of olive mill wastewater isolate *Delfina tsuruhatensis* in a combined process of pre-ozonation followed by treatment with SBP capsules, in increasing the biodegradation rate. This was the first study demonstrating synergy between the AOP treatment and subsequent biological SBP technology.

Microorganisms degrade steroidal hormones via two possible mechanisms: growth-linked (metabolic) and non-growth-linked (co-metabolic). Co-metabolic: heterotrophic and nitrifying bacteria) [13]. Only a few types of bacteria have been reported to degrade EE2 when it is added as the sole carbon and energy source: *Sphingobacterium sp. JCR5* [14], *R. zopfii*, and *Rhodococcus equi* [15]. The minimal half-life of EE2 in natural systems is several days. However, its half-life in WWTPs is much shorter, with its concentration being halved within hours, suggesting that the rate of the EE2 biodegradation is affected by bacterial concentration and diversity [13]. These results emphasize the potential of biodegradation treatment for EDCs and other micropollutants. *Rhodococcus* bacteria are defined as Gram-positive to Gram-variable, aerobic, and non-motile. Their cell morphology varies during different stages of their growth cycle. These bacteria grow in filaments and exhibit extensive branching and hyphal growth before fragmenting into irregular rod-shaped or coccoid units. Some strains can be found as cocci that later turn into short rods, whereas others continue transforming into long filamentous rods or start branching out in an elementary or extensive fashion. In this present work, *Rhodococcus* encapsulated SBP capsules were used to study the biodegradation of EE2.

In this study, nano-MgO_2_ is used for the degradation of EE2 under LP-UV light for the first time. We evaluate the role of MgO_2_ in photocatalytic degradation, SBP capsule-based biodegradation using *Rhodococcus zopfii*, and their synergetic effect on the degradation of EE2. *R. zopfii* is a well-known bacterium that specifically degrades estrogens. The production of reactive oxygen species (ROS) by an aqueous suspension of MgO_2_ is investigated under ambient conditions and under LP-UV light. This work targets the potential of metal peroxides in designing water-treatment systems for the degradation of persistent organic pollutants in the effluent, and the synergy with a novel biotreatment technology.

## 2. Materials and Methods 

### 2.1. Material Characterization 

X-ray powder diffraction measurements were performed on a D8 Advance diffractometer (Bruker AXS, Karlsruhe, Germany) with a secondary graphite monochromator and 2° Soller slits. UV-visible (Vis) spectra were recorded at room temperature using a Jasco V 570 UV-Vis spectrophotometer, and X-ray photoelectron spectroscopic (XPS) analysis was done using the K-Alpha instrument (XPS K-Alpha surface analysis, Thermo Fisher Scientific, Leeds, UK). Brunauer-Emmett-Teller (BET) surface area, pore volume, and radius were determined by N2 adsorption–desorption isotherms collected at 77 K using a Quantachrome instrument (Anton Paar, Graz, Austria). The morphology of the SBP capsule membrane was investigated using a high resolution scanning electron microscope (HRSEM, Zeiss Ultra Plus, Jena, Germany) equipped with a high-resolution field emission gun (FEG), operated at an accelerating voltage of 4 kV in a 3–5 nm working distance.

### 2.2. Analytical Procedures 

EE2 was analyzed by HPLC/UV (Agilent 1100 series instrument, Agilient Technologies, Waldbronn, Germany, using a Kinetex 2.6 µm EVO C18 column, 100 × 3 mm, with mobile-phase composition of 55% water and 45% acetonitrile (*v*/*v*), in isocratic mode. p-chloro benzene (pCBA) was analyzed using a Reprospher (Dr.Maisch GmbH, Ammerbuch-Entringen, Germany) 100 C8-DE 3 µm column, 100 × 3 mm. The pump was set for an isocratic program of 40% water (*v*/*v*), 60% MeOH (*v*/*v*), and 0.1% formic acid (*v*/*v*) at a flow rate of 0.5 mL/min. The injection volume was 10 µL and the detector wavelength was set to 243 nm.

### 2.3. Photocatalysis Experiments

The photocatalysis experiments were performed with a bench-scale LP-UV collimated-beam apparatus using a 43 W LP-UV lamp (Trojan Technologies Inc, London, ON, Canada) which emits monochromatic irradiation at 254 nm. Average irradiance was calculated using the spectral irradiance measured by a calibrated spectroradiometer (USB4000, Ocean Optics, Largo, FL, USA) placed in the same x–y position as the center of the crystallization dish and at the surface of the liquid suspension. The sample surface reflection, water absorbance, and petri factor were also considered in the calculation. Absorbance was recorded by UV-Vis spectrophotometer (Cary Bio100, Varian Inc., Palo Alto, CA, USA). The UV dose was calculated by multiplying the average irradiance by time of exposure [16].

### 2.4. Estimation of H_2_O_2_ by Potassium Permanganate (KMnO_4_) Titration

H_2_O_2_ produced from an aqueous suspension of MgO_2_ was measured by KMnO_4_ redox titration [8]. MgO_2_ (10 mg) was added to 100 mL water. Then 2 mL of 2 mM KMnO_4_ and 16 µL of 3 mM sulfuric acid (H_2_SO_4_) were added and the suspension was stirred under ambient conditions. At regular intervals, a 2mL aliquot of the sample was taken and filtered through a 0.44µm membrane. The H_2_O_2_ produced from the catalyst was estimated by titrating the filtered aliquot with known amounts of H_2_O_2_. Water without MgO_2_ was used as a control.

### 2.5. Colorimetric Estimation of H_2_O_2_ Using Strips

H_2_O_2_ produced from the catalyst was also estimated by peroxide test strips (Mquant, Merck, Germany). MgO_2_ (10 mg) was added to 100 mL water. The aqueous suspension was stirred under ambient conditions. At regular intervals, 2 mL of the sample were taken and centrifuged at 10,000 rpm and the color change of the strip dipped in the supernatant was observed. The concentration of H_2_O_2_ produced from aqueous suspensions of MgO_2_ was calculated from the color reference provided by the company. Water without MgO_2_ was used as a control.

### 2.6. Estimation of ·OH Radicals

The concentration of ·OH released from aqueous suspensions of MgO_2_ was analyzed indirectly using pCBA as a probe. The kinetics of ·OH production was directly correlated to the degradation of pCBA. The concentration of ·OH was estimated according to [17]:

(1) $$-\frac{d\left[\mathrm{pCBA}\right]}{\mathrm{dt}}=k_{\mathrm{OH}\;\;\mathrm{pCBA}}\;{\left[\cdot \mathrm{OH}\right]}_{\mathrm{ss}}$$

where K_OH_ pCBA is the rate constant of pCBA with ·OH, 5 × 109 M^−1^ s^−1^ at ambient temperature and pressure, and ·OH is the steady-state concentration of hydroxyl radicals.

MgO_2_ (10 mg) was added to 100 mL of 5 ppm pCBA in water and the aqueous suspension was exposed to LP-UV irradiation for 120 min. The samples were withdrawn at 30 min intervals and the amount of pCBA in solution was analyzed by HPLC/UV.

### 2.7. Photocatalytic Degradation of EE2

MgO_2_ (25 mg) was added to 100 mL of 3 ppm EE2 in 10 mM phosphate buffer and exposed to LP-UV irradiation for 60 min under constant stirring. At regular intervals, 1 mL of the sample was withdrawn and centrifuged at 10,000 rpm, and EE2 prior to and after treatment was analyzed by HPLC/UV. The rate of EE2 degradation was calculated as:

(2) $$\ln\frac{C_{t}}{C_{0}}=-kt$$

and percent removal of EE2 at time (t) was calculated as:

(3) $$\%\;\mathrm{Removal}=\frac{C_{0}-C_{t}}{C_{t}}\times 100$$

where C_0_ and C_t_ (mg/L) are the initial concentration of EE2 and the concentration of EE2 at time (t), respectively, and k is the rate of degradation (1/min).

### 2.8. Encapsulation of R. zopfii in Membrane-Based Capsules

Bacterial cultures were encapsulated in SBP capsules. The encapsulation procedure and experimental validation are detailed in patent PCT/IL2010/00256 [18], Menashe and Kurzbaum [9], and Azaizeh et al. [10]. Briefly, the bacterial cultures were encapsulated in cellulose-acetate microfiltration membrane capsules (2.5 cm long and 0.8 cm in diameter). This membrane creates a protected 3D space for the bacterial suspension. EE2-degrading *R. zopfii* (ATCC 51349) was purchased from the ATCC culture bank (Manassas, VA, USA). The bacterial strains were maintained on nutrient agar (Neogen, Lansing, MI, USA) and grown in nutrient broth (Neogen) with 5% (*w*/*v*) trehalose (Sigma, Rehovot, Israel) as a protective agent for the freeze-drying procedure. The cells were grown to an absorbance at 600 nm of 1 at 30 °C and 150 rpm, freeze-dried, and introduced into the cellulose-acetate membrane capsules under sterile conditions. At this stage the bacteria were inactive.

### 2.9. Activation of SBP Capsules

The capsules were washed with 70% EtOH for 1 min and placed in 300 mL of 5% (*w*/*v*) Luria Bertani medium (LB) in 10 mM phosphate buffer. The flask was closed with a breathing cap and incubated at 30 °C, 150 rpm for 5–7 days. Every 2–3 days, the capsules were moved to fresh 5% LB in 10 mM phosphate buffer.

### 2.10. Biodegradation of EE2 by SBP Capsules

Five activated capsules were taken from the Erlenmeyer flask, washed with 70% EtOH for 1 min and transferred to a 250 mL Erlenmeyer flask containing 3 ppm EE2 in 10 mM phosphate buffer (100 mL). The whole experiment was performed in triplicate and flasks were incubated at 30 °C at 150 rpm. At regular intervals, a 1 mL aliquot was collected from each bottle, centrifuged at 10,000 rpm, and the supernatant was analyzed by HPLC/UV to determine the EE2 concentration.

### 2.11. Photocatalytic Degradation of EE2 by MgO_2_ Followed by Biodegradation

MgO_2_ (25 mg) was added to 3 ppm EE2 in 10 mM phosphate buffer (100 mL) and exposed to LP-UV irradiation for 5, 15, and 25 min (corresponding to incident doses of 138, 414, 691 mJ /cm^2^). The treated aqueous suspensions were transferred to a sterile 250 mL Erlenmeyer flask. Five activated SBP capsules were added to each flask and the flasks were incubated at 30 °C at 150 rpm. Sample aliquots of 1 mL were taken at regular intervals throughout the experiment (photocatalysis and biodegradation), centrifuged at 10,000 rpm, and the supernatant was analyzed by HPLC/UV to determine the EE2 concentration.

### 2.12. EE2 Adsorption to the SBP Capsule Membrane

To evaluate the EE2 adsorption to the SBP capsule membrane, activated SBP capsules were sterilized by autoclaving prior to incubation in 250 mL Erlenmeyer flasks containing 100 mL of 10 mM phosphate buffer solution supplemented with 0.5 mg/L EE2. The sterile adsorption experiment was performed in triplicate with five sterile SBP capsules in each flask, while the control did not contain capsules. Samples for EE2 concentration determination were taken before and 24 h after capsule addition.

## 3. Results

### 3.1. Material Characterization 

**Figure 1:**
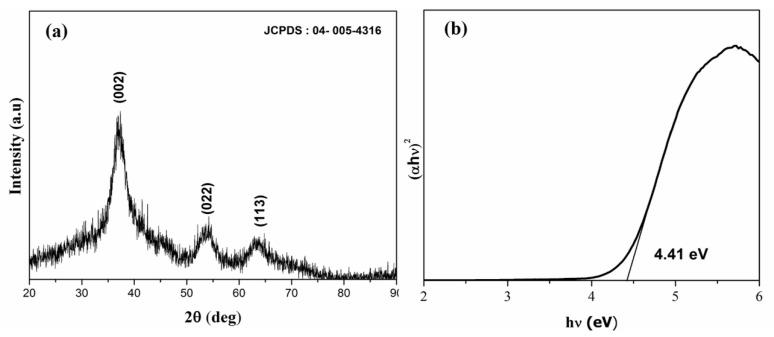
(**a**) X-ray diffractogram and (**b**) Tauc plot of MgO_2_.

The X-ray diffractogram (Figure 1a) confirmed the crystallization of pure MgO_2_ in the cubic phase with lattice parameter a = 4.839 Å and a space group of pa-3 (JCPDF-04-005-4316). The crystallite size was calculated from the Scherrer formula:

D = 0.9λ/β Cosθ(4)

where λ is 1.54060 Å, β is the full width at half maximum, and θ is the angle of diffraction. The calculated crystallite size was 1.76 nm. The optical band gap was determined from the UV-Vis absorption spectrum by Tauc plot (Figure 1b):

(αhν)n = A(hν − Eg)(5)

where α denotes the absorption coefficient, hν is the discrete photon energy, A is constant, Eg is the band gap, and the exponent n depends on the type of transition. The band gap was calculated to be 4.41 eV.

**Figure 2:**
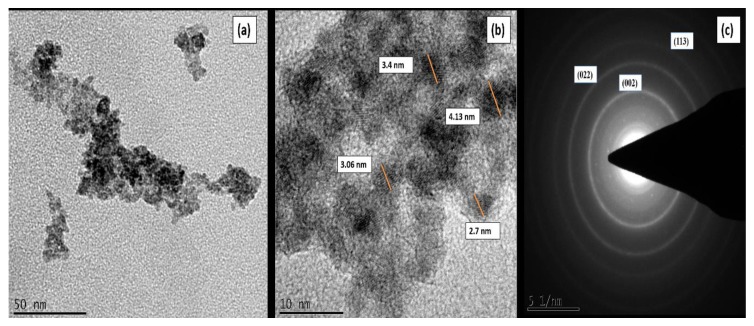
(**a**,**b**) HR-TEM of MgO_2_. (**c**) SAED of MgO_2_.

Figure 2 shows HR-TEM of MgO_2_. Figure 2a shows that synthesized MgO_2_ are highly agglomerated with nearly spherical like morphology. Figure 2b shows that the size of particles are in the range of 2.7–4.13 nm. SAED (Figure 2c) confirms that the synthesized catalyst is of nanocrytalline and shows planes corresponding to (002), (022), and (113), confirming the MgO_2_ phase.

**Figure 3:**
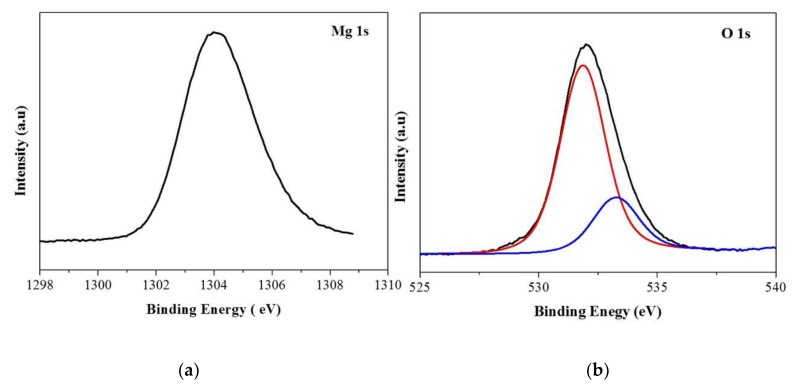
XPS of MgO_2_ (**a**) Mg 1s (**b**) O1s.

Figure 3 shows the XPS of MgO_2_. The binding energy of Mg1s at 1034 eV confirmed that Mg is in the +2 state (Figure 3a). O1s of MgO_2_ showed two peaks: the peak at the higher binding energy, 532.5 eV, corresponds to O_2_^2−^ species and the peak at 531.2 eV corresponds to hydroxyl species which are associated with oxygen vacancy, confirming the presence of peroxide species and oxygen vacancies on the surface of MgO_2_ (Figure 3b) [19]. The surface area of MgO_2_ determined by N_2_ adsorption–desorption isotherms was 308.32 m^2^/g. Measured pore volume and pore size were 0.497 cc/g and 8.07 nm, respectively (Figure S1). The results confirm MgO_2_ as a nanoscale photocatalyst with high specific surface area, resulting in more active sites for surface reaction.

### 3.2. ROS from Aqueous Suspensions of MgO_2_

**Figure 4:**
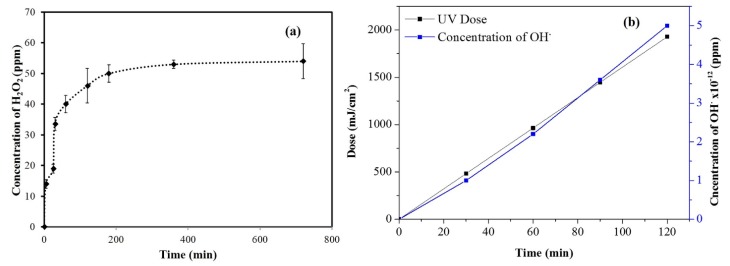
(**a**) H_2_O_2_ produced from aqueous suspensions of MgO_2_ under ambient laboratory conditions. (**b**) OH‧ radicals produced from aqueous suspensions of MgO_2_ under LP-UV irradiation.

Figure 4a shows the kinetics of H_2_O_2_ production from aqueous suspensions of MgO_2_ under ambient laboratory conditions calculated by KMnO_4_ titration. At initial time intervals of 0–30 min, H_2_O_2_-production rate increased; however, after 30 min, it decreased. By KMnO_4_ titration, the aqueous suspension of MgO_2_ (100 ppm) produced 54 ppm H_2_O_2_ after 12 h. Production of H_2_O_2_ from the catalyst was also confirmed by using colorimetric strips from Merck, which turn from white to blue when they react with H_2_O_2_. The color intensity is directly proportional to the concentration of H_2_O_2_. Table 1 shows the concentration of H_2_O_2_ produced from MgO_2_ as analyzed by colorimetric strips. These experiments suggested that aqueous suspensions of MgO_2_ produce H_2_O_2_, even in the absence of irradiation. Figure 4b shows the production of hydroxyl radicals from aqueous suspensions of MgO_2_ under LP-UV irradiation. In the dark, there was no degradation of pCBA, implying that OH**^‧^**radicals were not produced. Under LP-UV irradiation, there was a gradual increase in the production of OH‧ radicals with time and UV dose, reaching 5 × 10^−12^ M after 60 min.

### 3.3. Photocatalytic Degradation of EE2

Under ambient conditions without UV light, limited or no degradation of EE2 was observed by MgO_2_, ruling out absorption as the mechanism of EE2 removal from the water. Figure S2a shows the effect of MgO_2_ concentration on photocatalytic degradation of EE2, the efficiency of which increased with the increasing MgO_2_ concentration. The rate of EE2 degradation for 50, 100, and 150 ppm MgO_2_ was 0.015, 0.02, and 0.06/min, respectively. The amount of ROS produced from the catalyst increased with MgO_2_ under UV light, resulting in a higher degradation efficiency of EE2.

**Figure 5:**
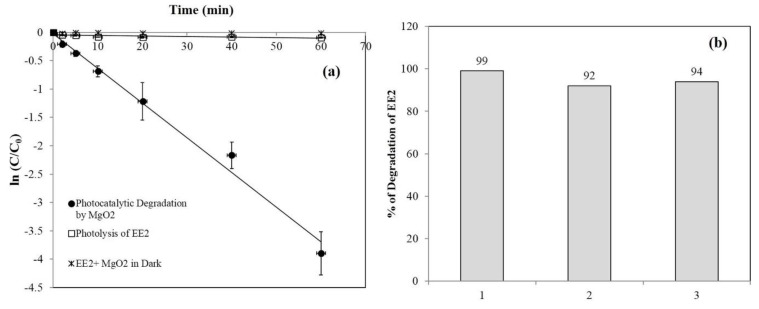
(**a**) Kinetics of photocatalytic and photolysis of 3 ppm EE2 in 10 mM phosphate buffer under LP-UV irradiation. (**b**) Reusability of MgO_2_ for degradation of 3 ppm EE2 in 10 mM phosphate buffer under LP-UV irradiation.

Figure 5a shows the photolytic and photocatalytic degradation of EE2 by 250 ppm MgO_2_. EE2 alone under LP-UV irradiation underwent 9% photolysis in 60 min, whereas in the presence of MgO_2_ and LP-UV irradiation, EE2 degradation reached 99% after 60 min. The rates of EE2 degradation by LP-UV and LP-UV/MgO_2_ were 0.001 and 0.06/min, respectively, implying that photocatalytic degradation by MgO_2_ plays a significant role in EE2 removal.

One of the main prerequisites for photocatalysts is reusability. After degrading EE2 for 60 min, the aqueous suspension of MgO_2_ was centrifuged and dried in an oven at 60 °C, and the catalyst was then reused for EE2 degradation (Figure 5b). The same catalyst was reused three times. In the first cycle, degradation of EE2 was 99%, and in the third cycle, it decreased to 94%. These results strongly affirm our claim that MgO_2_ can be reused for pollutant degradation.

Few reports exist on the degradation of pollutants by metal peroxides. Zhou et al. [20] investigated a CaO_2_/Fe^3+^ system for degradation of bisphenol A under ambient conditions; Lakshmi Prasanna and Vijayaraghavan [19] studied the degradation of rhodamine B using ZnO_2_/Fe^2+^ in the dark and under UV light, while Wu et al. [21] employed MgO_2_/Fe^2+^ for degradation of methylene blue under ambient conditions. However, this is the first investigation of aqueous suspensions of MgO_2_ for the degradation of EE2 under UV light.

The KMnO_4_ titrations and colorimetric strips suggested that aqueous suspensions of MgO_2_ produce H_2_O_2_ even under ambient conditions as:

MgO_2_ + H_2_O ↔ MgO + H_2_O_2_ (6)

Even though H_2_O_2_ was produced in the absence of irradiation, it could not degrade EE2. However, in the presence of UV light, H_2_O_2_ undergoes photolysis to produce ·OH (Equations (7)–(10)), degrading EE2 through oxidation. This is in agreement with the literature. Zhang et al. [22] and Zhang and Li [23] addressed the degradation of EE2 by H_2_O_2_ under UV light (254 nm) and observed that in the dark, H_2_O_2_ does not degrade EE2, but under UV light, H_2_O_2_ degrades EE2 via oxidation through OH‧ radicals:

H_2_O_2_ + hν ⟶ 2 ·OH (7)

·OH + H_2_O_2_ ⟶ H_2_O + ·HO_2_ (8)

·HO_2_ + H_2_O_2_ ⟶ ·OH + H_2_O + O_2_ (9)

2 ·HO_2_ ⟶ H_2_O_2_ + O_2_ (10)

The band gap of MgO_2_ is 4.41 eV and under LP-UV irradiation (at 254 nm), electrons (e-) are excited from the valence band (VB) to the conduction band (CB), leaving holes (h^+^) in the VB. Dissolved oxygen and water react with O_2_ and h+ to produce ROS, as shown in Equations (12)–(16);

MgO_2_ + hν ⟶ e^−^ (CB) + h^+^ (VB) (11)

H_2_O + h^+^ ⟶ ·OH + H^+^ (12)

O_2_ + e^−^ ⟶ ·O_2_^−^ (13)

·O_2_^−^ + H^+^ ⟶ ·HO_2_ (14)

·HO_2_ + H^+^ + e^−^ ⟶ H_2_O_2_ (15)

2 ·OH ⟶ H_2_O_2_ (16)

Thus, ROS are produced by the photolysis of H_2_O_2_ (Equations (7)–(10)), and due to the semiconductive mechanism of MgO_2_ (as shown in Equations (11)–(16)) under LP-UV light, they can successfully degrade EE2.

Since MgO_2_ produces H_2_O_2_ under ambient laboratory conditions, as shown in Equation (6), in the presence of Fe^2+^, H_2_O_2_ reacts and produces ROS as shown in Equations (17)–(19). To investigate the Fenton-like degradation by the MgO_2_/Fe^2+^ system under ambient conditions, we performed an experiment with 25 mg of MgO_2_ and 10 mg of Fe^2+^ added to 10 ppm EE2 and stirred under ambient conditions, and the efficiency of degradation was compared to that with 30% commercial H_2_O_2_. Samples (1 mL) were collected at various intervals and centrifuged, and EE2 was analyzed by HPLC/UV prior to and after treatment. Figure S2b shows the Fenton-like degradation by the MgO_2_/Fe^2+^ system and 30% H_2_O_2_ (100 µL)/Fe^2+^. Neither MgO_2_ nor H_2_O_2_ alone could degrade EE2 under ambient conditions, whereas in the presence of Fe^2+^, marked degradation was observed: 100% degradation of 10 ppm EE2 with the MgO_2_/Fe^2+^ system implied that the Fenton-like degradation shows higher kinetics of degradation than MgO_2_/LP-UV. However, even though the Fenton-like degradation by MgO_2_ was faster than that by MgO_2_/LP-UV, MgO_2_/Fe^2+^ creates iron sludge which we could not separate from the catalyst, preventing its reuse.

H_2_O_2_ + Fe^2+^ ⟶ ·OH + OH^−^ + Fe^3+^ (17)

Fe^3+^ + H_2_O_2_ ⟶ Fe^2+^ + ·HO_2_ + H^+^ (18)

Fe^2+^ + ·OH ⟶ Fe^3+^ + OH^−^ (19)

### 3.4. Biodegradation of EE2 by SBP Capsules

**Figure 6:**
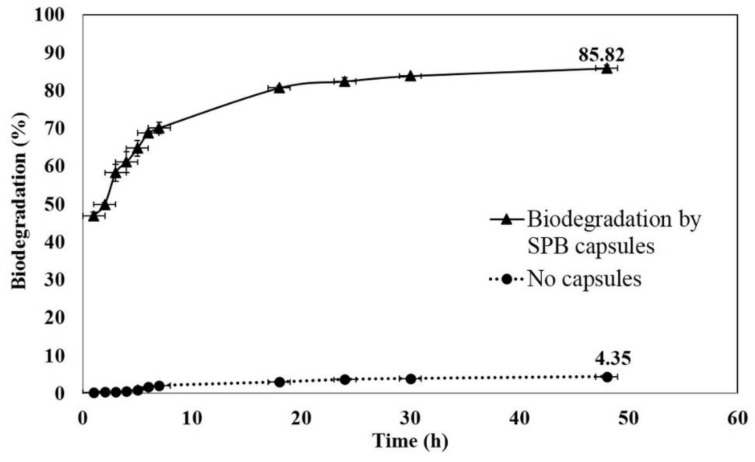
Biodegradation of 3 ppm EE2 in 10 mM phosphate buffer by *R. zopfii*-containing SBP capsules.

We encapsulated *R. zopfii* in the SBP capsules to evaluate EE2-biodegradation rate in two stages: as a standalone biotreatment and combined with MgO_2_/LP-UV. Figure 6 shows the biodegradation of 3 ppm EE2 by SBP-encapsulated *R. zopfii*. The rate of degradation was high during the initial time interval of 1–18 h, then plateaued after 24 h. Percent degradation of 3 ppm EE2 at 1, 7, 18, and 24 h was 47, 70, 81, and 82, respectively. However, after 24 h, only limited biodegradation was observed. Percent removal of EE2 at 30 °C in the sterile control experiment was 4.35, implying that EE2 is relatively stable and suggesting that the biodegradation by SBP capsules plays a major role in the removal of EE2 when it is the only process.

In the sterile adsorption experiment, the control system without SBP capsules showed no change in the EE2 concentration during the experimental interval. This was also true for the five sterile SBP capsules, which did not alter the EE2 concentration in the solution (*t*-test, *p* < 0.05, *n* = 6) (data not shown). Thus the capsules’ cellulose-acetate membrane and other internal components (dead biomass) have no affinity for the EE2 molecules, and the reduction in EE2 concentration due to passive adsorption is negligible.

**Figure 7:**
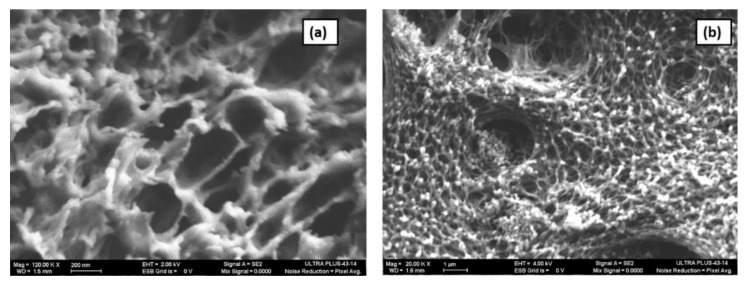
(**a**) Outer surface of the SBP capsule. (**b**) Cross-section of SBP capsule membrane.

Figure 7 shows HR-SEM of the SBP capsule membrane’s outer structure and cross-section perpendicular to the capsule plane. The outer surface of the capsule is a mesh-like structure with nanopores (Figure 7a) and the cross-section at higher magnification (120 k X) shows the presence of nanopores with diameter ranging from 50 to 300 nm. This unique membrane structure is assumed to allow molecule trafficking, not only through the membrane pores, but also across the entire capsule surface. As previously indicated by Kurzbaum et al. [11], this might be the reason for the phenols’ high trafficking rate and consequent rapid biodegradation rate.

In other encapsulation techniques, the microbial culture is embedded in a polymer matrix such as sodium alginate and cellulose-based gel [24,25], Owing to the low availability of nutrients and oxygen due to poor diffusion rates, the bacteria show low biodegradation efficiency. In our case, the membrane is designed to enable the diffusion of nutrients, pollutants, and oxygen for enhanced biodegradation efficiency. In the usual bioaugmentation process, where suspended cultures are used, loss or dilution of biomass occurs due to the continuous inflow and outflow of water, resulting in reduced biodegradation efficiency of the biomass. Therefore, it is essential to protect the biomass inside the reactor to achieve maximum efficiency. In our case, the SBP capsule membrane protects the biomass by preventing culture dilution, thereby enhancing the biodegradation efficiency. *R. zopfii* is rod-shaped bacterium that is 1.1–2.85 µm long and 0.55–0.8 µm wide [26]. Since the pore sizes of the SBP capsule membrane Figure 7a,b is smaller than the bacterium, the membrane prevents the escape of the bacteria from the capsule. On the other hand, these membrane pores facilitate the diffusion of contaminants, nutrients, and metabolites into and out of the capsule. EE2 from the medium diffuses into the capsule through the pores and the suspended bacteria inside the capsule degrade it; then, the metabolites diffuse back into the reactor medium. This constant diffusion of EE2 and metabolites plays a significant role in the biodegradation of EE2.

Larcher and Yargeau [13] isolated *Rhodococcus* species from activated sludge and studied the biodegradation of 5 ppm EE2. Percent degradation by suspended cultures of *R. equi, R. erythropolis, R. rhodochrous and R. zopfii* after 300 h were 61, 46, 100, and 38, respectively. Yu et al. [27] also isolated *Rhodococcus* cultures from activated sludge and studied the biodegradation of 3 ppm EE2; they observed 100% degradation after 3 days. O’Grady et al. [28] studied the biodegradation of 1.4 ppm EE2 and observed 47% degradation after 13 h in the presence of co-substrate. These results suggest that biodegradation of EE2 by SBP-encapsulated *R. zopfii*, where significant removal of EE2 was observed within 24 h, is relatively more efficient than that by suspended *Rhodococcus* cultures, as reported in the literature. This strengthens the concept that the SBP membrane allows the rapid diffusion of molecules across it, and combined with the suspended form of the encapsulated bacteria within the confined environment of the capsules, results in high biodegradation rates.

### 3.5. Photocatalytic Degradation of EE2 by MgO2/LP-UV Followed by Biodegradation by SBP Capsules

**Figure 8:**
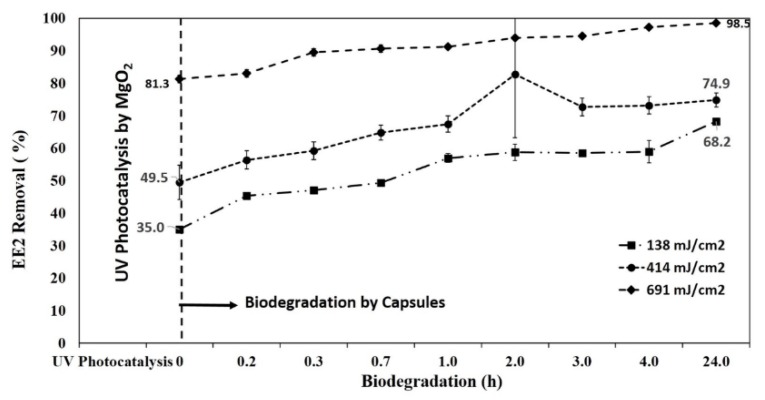
Sequential photocatalysis–biodegradation of 3 ppm EE2 in 10 mm phosphate buffer.

A successive combined process involving different irradiation times in the presence of catalyst followed by a biological stage using SBP capsules was performed to evaluate the effect of the photocatalytic step on EE2 biodegradability. Figure 8 illustrates the synergetic effect of photocatalysis and biodegradation of 3 ppm EE2. EE2 was exposed to various doses of LP-UV irradiation in the presence of MgO_2_ and then subjected to biodegradation by SBP capsules at 300 C. Percent degradation of EE2 for the samples exposed to doses of 138, 414, 691 mJ/cm^2^ in the presence of MgO_2_ was 35, 50, and 81, respectively. Five *R. zopfii*-containing SBP capsules were added to each UV-irradiated EE2 sample to understand the effect of biodegradation. EE2 was the sole carbon source for the encapsulated *R. zopfii*, and therefore complete degradation was expected.

Table 2 gives the percent biodegradation of EE2 samples exposed to MgO_2_/LP-UV followed by biodegradation by SBP capsules for various time intervals. It is clear that SBP biotreatment is effective because used alone, it reduced EE2 concentration by 61% during the first 4 h of incubation.

However, our study aimed to provide a much more effective treatment tool by integrating AOP and biotreatment. MgO_2_/LP-UV, with irradiation for 25 min (691 mJ/cm^2^), removed 81% of EE2. When the irradiated solution was then exposed to the SBP capsules for 4 h, 97% biodegradation was observed. Without prior photocatalytic treatment, it took 24 h for the SBP capsules to remove 82.34% of EE2. Figure S3 shows sequential photolysis by LP-UV irradiation and biodegradation of EE2 for 24 h.

AOP is an effective treatment, especially with respect to cleaving double bonds and rings [29]. Double bonds and ring cleavage are hard to challenge by biological digestive systems. Therefore, the observed synergy between the two systems (MgO_2_/LP-UV and biological) stems from the fact that the MgO_2_/LP-UV pre-treatment renders the EE2 molecules more susceptible to the biological systems, resulting in reduced biodegradation time. Biodegradation alone was only able to remove 61% EE2 in 4 h; however, when combined with the photocatalytic treatment, its contribution of biodegradation by SBP capsules decreased up to 24% (at the lower dose of 138 mJ/cm^2^). MgO_2_/LP-UV as the sole treatment presented 81% EE2 biodegradation at 691 mJ/cm^2^. Thus, the combined treatment offers a rapid-tech treatment potential that is only hinted at in this study. Research is needed to gather more evidence on combined physicochemical and biological treatments. It is essential to find the right combination of AOP and biological treatment, since the biotreatment requires less energy and cost investment.

Li et al. [30] investigated the combined photocatalysis-biodegradation of 2, 4, 5-trichlorophenol by a TiO_2_-coated biofilm composed of various bacterial strains of *Ralstonia, Bradyrhizobium, Methylobacterium, Cupriavidus*, and *Pandoraea* and found an enhanced degradation and mineralization of the pollutant under UV irradiation owing to photocatatalytic degradation and mineralization by TiO_2_, and biodegradation by the biofilm coated on a sponge carrier. Zhang et al. [31] studied simultaneous photodegradation and biodegradation by g-C_3_N_4_-P25 and photosynthetic bacteria encapsulated in sodium-alginate beads, and observed significantly enhanced degradation and mineralization of the pollutants and reduced chemical oxygen demand of synthetic wastewater composed of textile dyes. Our future objective in this project is to coat MgO_2_ on cellulose-based *R. zopfii*-containing capsules and study simultaneous photocatalysis and biodegradation of EE2 and various other pollutants in wastewater.

A pilot plant based on the photocatalysis-biodegradation process was set up at a local hospital to treat 0.5 ppm of EE2 in simulated wastewater and real hospital wastewater samples. In the future, sequential photocatalysis-biodegradation will be demonstrated on site. This sequential treatment is expected to reduce the LP-UV consumption and the hydraulic residence time for biodegradation. Research is needed to obtain a cost-effective balance between the LP-UV pre-treatment and the SBP biotreatment.

## 4. Conclusions

The photocatalytic degradation of EE2 by aqueous suspensions of nano MgO_2_ was investigated for the first time. ROS produced from aqueous suspensions of catalyst under LP-UV plays a significant role in the degradation of EE2. The biodegradation of EE2 by bacteria encapsulated SBP capsules was also investigated. Even though biodegradation was effective in the removal of EE2, it is a time consuming process. However, the combination with photocatalytic degradation reduces the load on biodegradation significantly. Effective combinations of UV treatment and biodegradation produced a greater level of EE2 degradation at a lower UV dose and significantly less biodegradation time than either treatment used separately. Another potential advantage that was not studied here is the impact of this treatment on transformation products and the advantage of selective biomass for by-product removal.

## Figures and Tables

**Table 1:** Concentration of H_2_O_2_ produced by aqueous suspensions of MgO_2_ as analyzed by colorimetric Strips.

Time (min)	Concentration of H_2_O_2_ (ppm)
0	0.2–5
5	0.2–5
25	0.4–10
35	20–30
60	20–30

**Table 2:** Biodegradation of 3 ppm EE2 by SBP capsules after photocatalytic treatment by MgO_2_.

	% Biodegradation of EE2 at the Specified Time (h)			
Dose (mJ/cm^2^)	0	1	3	4
0	0	47	58	61
138	35	57	58	59
414	50	67	72	73
691	81	91	94	97

## Supplementary Materials

The following are available online at https://www.mdpi.com/1996-1944/13/1/83/s1, Figure S1: Nitrogen adsorption-desorption isotherms of nano MgO_2_, Figure S2: (a) Effect of concentration ofMgO_2_ on degradation of 3 ppm EE2 under UV-LP, (b) Fenton-like degradation of 10 ppm EE2 by MgO_2_/ Fe^2+^ and H_2_O_2_/ Fe^2+^ system, Figure S3: Sequential Photolysis by LP-UV and biodegradation of EE2.

## References

[1] Adeel M., Song X., Wang Y., Francis D., Yang Y. (2017). Environmental impact of estrogens on human, animal and plant life: A critical review. Environ. Int..

[2] Sarkar S., Ali S., Rehmann L., Nakhla G., Ray M.B. (2014). Degradation of estrone in water and wastewater by various advanced oxidation processes. J. Hazard. Mater..

[3] Zhang X., Chen P., Wu F., Deng N., Liu J., Fang T. (2006). Degradation of 17α-ethinylestradiol in aqueous solution by ozonation. J. Hazard. Mater..

[4] Rosenfeldt E.J., Linden K.G. (2004). Degradation of endocrine disrupting chemicals bisphenol A, ethinyl estradiol, and estradiol during UV photolysis and advanced oxidation processes. Environ. Sci. Technol..

[5] Frontistis Z., Xekoukoulotakis N.P., Hapeshi E., Venieri D., Fatta-Kassinos D., Mantzavinos D. (2011). Fast degradation of estrogen hormones in environmental matrices by photo-Fenton oxidation under simulated solar radiation. Chem. Eng. J..

[6] Shappell N.W., Vrabel M.A., Madsen P.J., Harrington G., Billey L.O., Hakk H., Larsen G.L., Beach E.S., Horwitz C.P., Ro K. (2008). Destruction of estrogens using Fe-TAML/peroxide catalysis. Environ. Sci. Technol..

[7] Krishnan S., Rawindran H., Sinnathambi C.M., Lim J.W. (2017). Comparison of various advanced oxidation processes used in remediation of industrial wastewater laden with recalcitrant pollutants. IOP Conf. Ser. Mater. Sci. Eng..

[8] Wolanov Y., Prikhodchenko P.V., Medvedev A.G., Pedahzur R., Lev O. (2013). Zinc dioxide nanoparticulates: A hydrogen peroxide source at moderate pH. Environ. Sci. Technol..

[9] Menashe O., Kurzbaum E. (2014). Small-bioreactor platform technology as a municipal wastewater additive treatment. Water Sci. Technol..

[10] Azaizeh H., Kurzbaum E., Said O., Jaradat H., Menashe O. (2015). The potential of autochthonous microbial culture encapsulation in a confined environment for phenol biodegradation. Environ. Sci. Pollut. Res..

[11] Kurzbaum E., Raizner Y., Cohen O., Suckeveriene R.Y., Kulikov A., Hakimi B., Iasur Kruh L., Armon R., Farber Y., Menashe O. (2017). Encapsulated pseudomonas putida for phenol biodegradation: Use of a structural membrane for construction of a well-organized confined particle. Water Res..

[12] Bar Oz Y., Mamane H., Menashe O., Cohen-Yaniv V., Kumar R., Iasur Kruh L., Kurzbaum E. (2018). Treatment of olive mill wastewater using ozonation followed by an encapsulated acclimated biomass. J. Environ. Chem. Eng..

[13] Larcher S., Yargeau V. (2013). Biodegradation of 17α-ethinylestradiol by heterotrophic bacteria. Environ. Pollut..

[14] Haiyan R., Shulan J., ud din Ahmad N., Dao W., Chengwu C. (2007). Degradation characteristics and metabolic pathway of 17α-ethynylestradiol by Sphingobacterium sp. JCR5. Chemosphere.

[15] Yoshimoto T., Nagai F., Fujimoto J., Watanabe K., Mizukoshi H., Makino T., Kimura K., Saino H., Sawada H., Omura H. (2004). Degradation of estrogens by Rhodococcus zopfii and Rhodococcus equi isolates from activated sludge in wastewater treatment plants. Appl. Environ. Microbiol..

[16] Taylor-Edmonds L., Lichi T., Rotstein-Mayer A., Mamane H. (2015). The impact of dose, irradiance and growth conditions on Aspergillus niger (renamed A. brasiliensis) spores low-pressure (LP) UV inactivation. J. Environ. Sci. Heal. Part A Toxic/Hazardous Subst. Environ. Eng..

[17] Cho M., Chung H., Choi W., Yoon J. (2004). Linear correlation between inactivation of E. coli and OH radical concentration in TiO_2_ photocatalytic disinfection. Water Res..

[18] Menashe O. (2010). Microorganism comprising particles and uses of same. Patent.

[19] Lakshmi Prasanna V., Vijayaraghavan R. (2017). Simultaneous fenton-photocatalytic reactions through a new single catalyst (nano ZnO_2_ /Fe^2+^) for dye degradation. J. Phys. Chem. C.

[20] Zhou Y., Fang X., Wang T., Hu Y., Lu J. (2017). Chelating agents enhanced CaO_2_ oxidation of bisphenol a catalyzed by Fe^3+^ and reuse of ferric sludge as a source of catalyst. Chem. Eng. J..

[21] Wu D., Bai Y., Wang W., Xia H., Tan F., Zhang S., Su B., Wang X., Qiao X., Wong P.K. (2019). Highly pure MgO_2_ nanoparticles as robust solid oxidant for enhanced Fenton-like degradation of organic contaminants. J. Hazard. Mater..

[22] Zhang Z., Feng Y., Liu Y., Sun Q., Gao P., Ren N. (2010). Kinetic degradation model and estrogenicity changes of EE2 (17α-ethinylestradiol) in aqueous solution by UV and UV/H_2_O_2_ technology. J. Hazard. Mater..

[23] Zhang A., Li Y. (2014). Removal of phenolic endocrine disrupting compounds from waste activated sludge using UV, H_2_O_2_, and UV/H_2_O_2_ oxidation processes: Effects of reaction conditions and sludge matrix. Sci. Total Environ..

[24] Duan L., Wang H., Sun Y., Xie X. (2016). Biodegradation of phenol from wastewater by microorganism immobilized in bentonite and carboxymethyl cellulose gel. Chem. Eng. Commun..

[25] González G., Herrera G., García M.T., Peña M. (2001). Biodegradation of phenolic industrial wastewater in a fluidized bed bioreactor with immobilized cells of Pseudomonas putida. Bioresour. Technol..

[26] Mohd-Towel R., Amir A., Abdul-Talib S. (2015). Physical Characterization of *Rhodococcus zopfii* DSM 44108 bacteria isolated from municipal sludge. Appl. Mech. Mater..

[27] Yu C.-P., Roh H., Chu K.-H. (2007). 17β-estradiol-degrading bacteria isolated from activated sludge. Environ. Sci. Technol..

[28] O’Grady D., Evangelista S., Yargeau V. (2009). Removal of Aqueous 17α-ethinylestradiol by Rhodococcus species. Environ. Eng. Sci..

[29] Oturan M.A., Aaron J. (2014). Advanced oxidation processes in water/wastewater treatment: Principles and applications. A review. Crit. Rev. Environ. Sci. Technol..

[30] Li G., Park S., Kang D.-W., Krajmalnik-Brown R., Rittmann B.E. (2011). 2,4,5-trichlorophenol degradation using a novel TiO_2_ -coated biofilm carrier: Roles of adsorption, photocatalysis, and biodegradation. Environ. Sci. Technol..

[31] Zhang X., Wu Y., Xiao G., Tang Z., Wang M., Liu F., Zhu X. (2017). Simultaneous photocatalytic and microbial degradation of dye-containing wastewater by a novel g-C_3_N_4_-P25/photosynthetic bacteria composite. PLoS ONE.

