# Supplementary material for: LP-UV-Nano MgO2 Pretreated Catalysis Followed by Small Bioreactor Platform Capsules Treatment for Superior Kinetic Degradation Performance of 17α-Ethynylestradiol
Source: Materials (Basel). 2019 Dec 23;13(1):83. doi: 10.3390/ma13010083 (PMC6982337; doi:10.3390/ma13010083)
Supplement: Supplementary file 1 [file materials-13-00083-s001.pdf]

Supporting Information

Article

LP-UV-nano MgO_2 pretreated catalysis followed by small platform bioreactor capsules treatment for superior kinetic degradation performance of 17α-Ethynylestradiol

Lakshmi Prasanna Vaddadi [1], Dror Avisar [1,*], Vinod Kumar Vadivel [2], Ofir Menashe [3], Eyal Kurzbaum [4,5], Vered Cohen-Yaniv [2] and Hadas Mamane [2]

[1] The Water Research Center, Hydrochemistry Laboratory, Porter School for Environment and Earth Sciences, Raymond and Beverly Sackler Faculty of Exact Sciences, Tel Aviv University, Tel Aviv 69978, Israel; lakshmip@mail.tau.ac.il

[2] The Water Research Center, Environmental Engineering Program, School of Mechanical Engineering, Faculty of Engineering, Tel Aviv University, Tel Aviv 69978, Israel; vinodkumarv@mail.tau.ac.il (V.K.V.); verver812@gmail.com (V.C.-Y.); hadasmg@tauex.tau.ac.il (H.M.)

[3] Water Industry Engineering Department, Achi Racov Engineering School, Kinneret College on the Sea of Galilee, M.P. Emek Ha'Yarden 15132, Israel; ofirmn@kinneret.ac.il

[4] Shamir Research Institute, University of Haifa, P.O. Box 97, Qatzrin 12900, Israel; ekurzbaum@univ.haifa.ac.il

[5] Department of Geography and Environmental Studies, University of Haifa, Mount Carmel, Haifa 3498838, Israel

* Correspondence: droravi@tauex.tau.ac.il; Tel.: +972-3-6405534

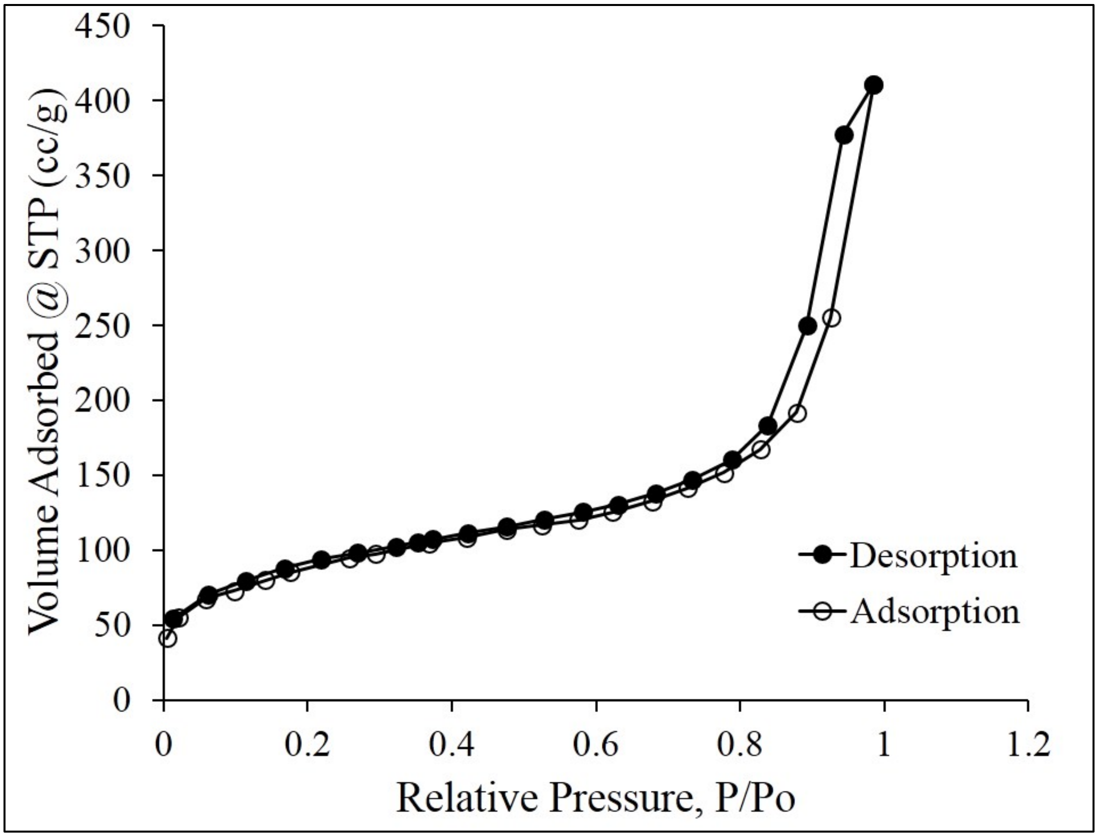

Figure S1. Nitrogen adsorption–desorption isotherms of nano-MgO_2.

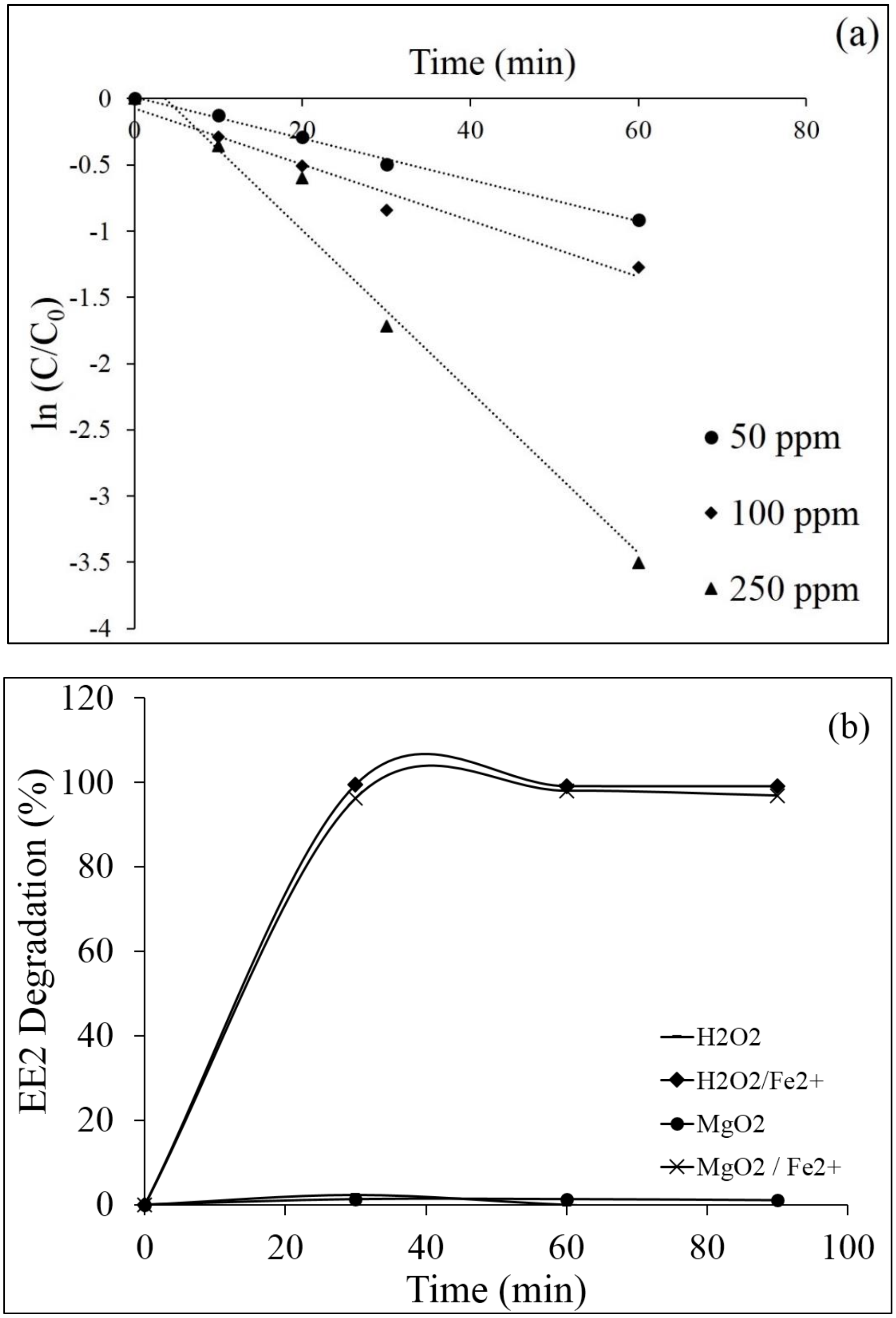

Figure S2. (a) Effect of MgO_2 concentration on degradation of 3 ppm EE2 under UV-LP irradiation. (b) Fenton-like degradation of 10 ppm EE2 by MgO_2/Fe^{2+} and H_2O_2/Fe^{2+} systems.

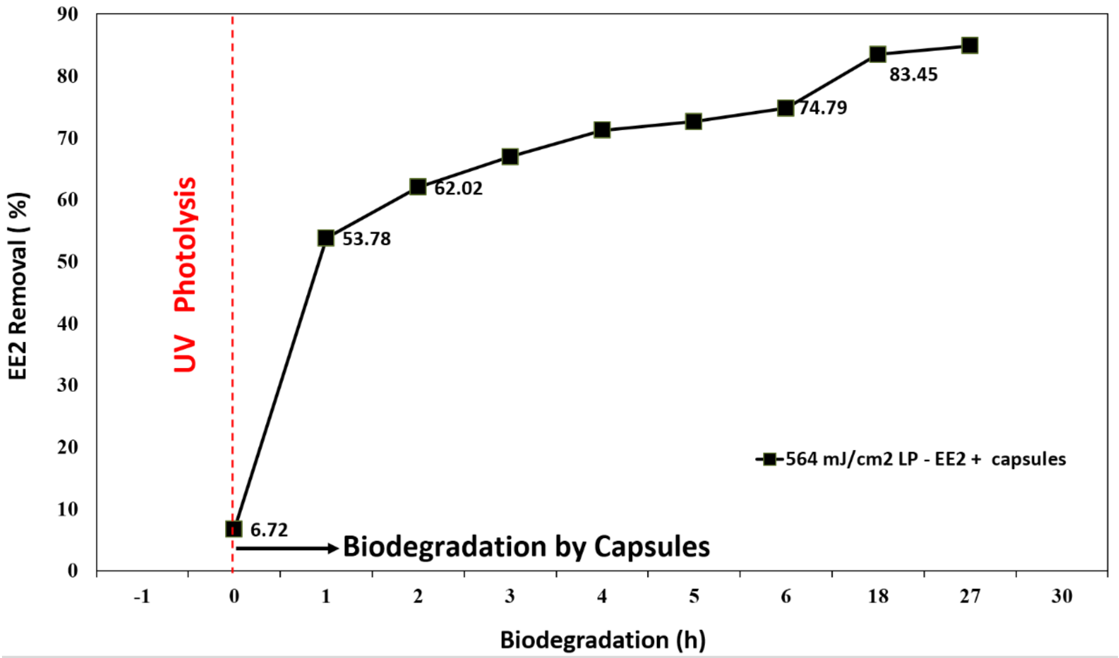

Figure S3. Sequential Photolytic degradation by LP-UV and biodegradation of EE2.
